# A Clinical Trial of Kampo Formulae for the Treatment of Symptoms of Yusho, a Poisoning Caused by Dioxins and Related Organochlorine Compounds

**DOI:** 10.1093/ecam/nep209

**Published:** 2011-01-09

**Authors:** Hiroshi Uchi, Shoji Tokunaga, Chikage Mitoma, Satoko Shibata, Naoki Hamada, Yoichi Nakanishi, Junboku Kajiwara, Takesumi Yoshimura, Masutaka Furue

**Affiliations:** ^1^Research and Clinical Center for Yusho and Dioxin, Kyushu University Hospital, Fukuoka 812-8582, Japan; ^2^Department of Dermatology, Graduate School of Medical Sciences, Kyushu University, Japan; ^3^Department of Medical Informatics, Kyushu University Hospital, Japan; ^4^Research Institute for Diseases of the Chest, Kyushu University Hospital, Japan; ^5^Fukuoka Institute of Health and Environmental Sciences, Fukuoka, Japan

## Abstract

The objective of this study was to evaluate the effectiveness of traditional herbal medicines (Kampo) on the symptoms of Yusho. Yusho is a mass food poisoning that was caused by ingestion of rice oil contaminated with dioxins and related organochlorines in 1968. Patients with Yusho suffer from skin symptoms (acneform eruptions, liability to suppuration and pigmentation), respiratory symptoms (cough and expectoration of sputum), neurological symptoms (numbness and paresthesia of extremities), arthralgia and general fatigue, and no effective treatment has yet been developed. In this clinical trial, four Kampo formulae (Bakumondo-to, Keigai-rengyo-to, Gosha-jinki-gan and Hochu-ekki-to) were administered to four representative Yusho symptoms (respiratory, skin, neurological symptoms and general fatigue), respectively. Twenty-seven Yusho patients were enrolled and two formulae were administered to each patient for half-a-year each. The effectiveness of Kampo formulae was estimated by changes in the intensity of symptoms measured by a visual analogue scale (VAS) of 100 mm recorded at baseline and after administration of each formula. The influence of Kampo formulae on patients' quality of life (QOL) was also assessed by the SF-36 (NBS). Twenty-five patients completed the treatment. Bakumondo-to significantly improved respiratory symptoms as well as patients' QOL in the context of vitality, compared with other formulae. In contrast, Hochu-ekki-to impaired patients' QOL in the context of physical functioning and vitality, compared with other formulae. This study demonstrated for the first time that a Kampo formula Bakumondo-to is useful for treating respiratory symptoms caused by dioxins.

## 1. Introduction

A mass food poisoning involving at least 1900 individuals occurred in western Japan in 1968, which was later referred to as Yusho oil disease because it was caused by the ingestion of rice bran oil contaminated with dioxins, polychlorinated biphenyls (PCBs) and other related organochlorine compounds [1–3]. Patients of Yusho presented various clinical symptoms, such as acneform eruptions, comedos, dark-brownish nail pigmentation, increased discharge from the meibomian glands, pigmentation of oral mucosa, numbness of extremities, cough with expectoration, articular swelling and arthralgia, irregular menstruation in women, general fatigue and stomachache [4]. Forty years have passed since the outbreak, but patients still suffer from many symptoms because no effective therapy for Yusho has been developed. Although blood levels of PCBs and dioxins in Yusho patients have been gradually decreasing during the 40-year period, recent studies showed that mean blood levels of total dioxins and 2,3,4,7,8-pentachlorinated dibenzofuran (penta-CDF), which is recognized as the most important causative agent for development of symptoms of Yusho, in Yusho patients from 2001 to 2003 were still 3.4–4.8 and 11.6–16.8 times higher, respectively, than those in normal controls [5]. Blood levels of dioxins in Yusho patients are known to be significantly correlated with the intensity of Yusho symptoms, such as skin symptoms, general fatigue, respiratory symptoms and neurological symptoms [6–8].

Kampo is a traditional herbal medicine that originated in China, and has been developed into a unique form in Japan. Each Kampo formula comprises several herbal extracts intended to increase the treatment effects and to diminish the adverse reactions of each herb, and is selected according to the pharmacological features and constitution of each patient [9, 10]. In this clinical trial, four Kampo formulae (Bakumondo-to, Keigai-rengyo-to, Hochu-ekki-to and Gosha-jinki-gan) were selected for the following four symptoms of Yusho, respectively: respiratory symptoms (cough and expectoration of sputum), skin symptoms (acneform eruptions, liability to suppuration and pigmentation), general fatigue and neurological symptoms (numbness and paresthesia of extremities). Bakumondo-to is widely used to treat chronic airway diseases such as bronchitis and asthma because of its antitussive effect [11, 12]. Keigai-rengyo-to is indicated for the treatment of chronic purulent diseases such as acne vulgaris because of its antibacterial activities [13, 14]. Hochu-ekki-to has long been used for the treatment of severe weakness in patients, especially those with a delicate constitution, caused by general fatigue or loss of appetite or ingestion [10]. Gosha-jinki-gan has been widely used for the treatment of diabetic neuropathy, urinary frequency and incontinence, and lower limb/lumbar pain [15]. The objective of this study was to determine the clinical effectiveness of these Kampo formulae for treatment of the symptoms of Yusho. The influence of Kampo formulae on patients' quality of life (QOL), laboratory values of blood, and blood concentrations of dioxins, PCBs and polychlorinated quaterphenyl (PCQ) was also assessed.

## 2. Methods

### 2.1. Subjects

Patients aged ≥20 years who fulfilled the diagnostic criteria for Yusho established by the National Study Group for the Therapy of Yusho were eligible for this study. Patients were not eligible for the study if they had serious diseases (malignant neoplasms, hepatic disorders, renal disorders or blood disorders), had been treated with any other Kampo formulae for more than four weeks prior to the study or were women with confirmed or suspected pregnancy, nursing women or women who desired to become pregnant during the trial. Patients who were judged to have an ineligible health condition by medical doctors were excluded. The trial protocol was approved by the institutional ethics committee of Kyushu University Hospital, and all patients gave their written informed consents.

### 2.2. Preparation of Kampo Formulae

Each Kampo formula was manufactured as a spray-dried powder of hot water extracts obtained from several medicinal plants. The daily dose of each formula was 7.5 g, except Bakumondo-to (9 g), and was administered orally three times a day before each meal. Nine grams of Bakumondo-to contained 6 g of extracts from 10 g enlarged part of the root of *Ophiopogon japonicus* Ker-Gawler, 5 g tuber of *Pinellia ternata* Breitenbach, 2 g root of *Panax ginseng* C.A. Meyer, 5 g seed of *Oryza sativa* Linne, 3 g fruit of *Zizyphus jujuba* Miller var. *intermis* Rehder, and 2 g root of *Glycyrrhiza uralensis* Fischer. Gosha-jinki-gan (7.5 g) contained 4.5 g of extracts from 5 g root of *Rehmannia glutinosa* Liboschitz var. *purpurea* Makino, 3 g rhizome of *Dioscorea japonica* Thunberg, 3 g fruit of *Cornus officinalis* Siebold et Zuccarini, 3 g Sclerotium of *Poria cocos* Wolf, 3 g rhizome of *Alisma orientale* Juzepczuk, 3 g root bark of *Paeonia suffruticosa* Andrews, 1 g bark of the trunk of *Cinnamomum cassia* Blume, 1 g tuber of *Aconitum japonicum* Thunberg, 3 g root of *Achyranthes fauriei* Leveille et Vaniot, and 3 g seed of *Plantago asiatica* Linne. Hochu-ekki-to (7.5 g) contained 5 g of extracts from 4 g root of *Panax ginseng* C.A. Meyer, 4 g root of *Astragalus membranaceus* Bunge, 4 g rhizome of *Atractylodes lancea* De Candolle, 2 g root of *Bupleurum falcatum* Linne, 3 g root of *Angelica acutiloba* Kitagawa, 1 g rhizome of *Cimicifuga simplex* Wormskjord, 2 g pericarp of the ripe fruit of *Citrus unshiu* Markovich, 0.5 g rhizome of *Zingiber officinale* Roscoe, 2 g fruit of *Zizyphus jujuba* Miller var. *intermis* Rehder, and 1.5 g root of *Glycyrrhiza uralensis* Fischer. 7.5 g of Keigai-rengyo-to contained 4.5 g of extracts from 1.5 g root of *Scutellaria baicalensis* Georgi, 1.5 g bark of *Phellodendron amurense* Ruprecht, 1.5 g rhizome of *Coptis japonica* Makino, 1.5 g root of *Platycodon grandiflorus* A. De Candolle, 1.5 g immature fruit of *Citrus aurantium* Linne var. *daidai* Makino, 1.5 g spike of *Schizonepeta tenuifolia* Briquet, 1.5 g root of *Bupleurum falcatum* Linne, 1.5 g fruit of *Gardenia jasminoides* Ellis, 1.5 g root of *Rehmannia glutinosa* Liboschitz var. *purpurea* Makino, 1.5 g root of *Paeonia lactiflora* Pallas, 1.5 g rhizome of *Cnidium officinale* Makino, 1.5 g root of *Angelica acutiloba* Kitagawa, 1.5 g terrestrial part of *Mentha arvensis* Linne var. *piperascens* Malinvaud, 1.5 g root of *Angelica dahurica* Bentham et Hooker, 1.5 g root of *Saposhnikovia divaricata* Schischkin, 1.5 g fruit of *Forsythia suspensa* Vahl, and 1 g root of *Glycyrrhiza uralensis* Fischer. All four Kampo formulae were generously supplied by Tsumura Co., Tokyo, Japan.

### 2.3. Endpoints

The primary endpoint was the intensity of symptoms measured on a visual analogue scale (VAS) of 100 mm. The target symptoms were general fatigue for Hochu-ekki-to, skin symptoms (acneform eruptions, liability to suppuration and pigmentation) for Keigai-rengyo-to, neurological symptoms (numbness and paresthesia of extremities) for Gosha-jinki-gan, and respiratory symptoms (cough and expectoration of sputum) for Bakumondo-to. Secondary endpoints were the intensity of other symptoms for which each Kampo formula was not specifically targeted, patients' QOL evaluated by the SF-36 (NBS) [16], laboratory values of blood, and blood concentrations of dioxins, PCBs and PCQ.

Primary and secondary endpoints were measured at baseline and after each treatment. Laboratory tests were additionally conducted when possible side effects were observed, or optionally with any other reasons. The VAS and SF-36 were measured by trained nurses who had experience in communicating with Yusho patients. A VAS was a horizontal line, 100 mm in length, and the left-hand end (0 mm) represented no symptom, whereas the right-hand end (100 mm) represented the severest symptom. At baseline and after each treatment, patients received a questionnaire having four lines of VAS for four symptoms. Trained nurses then asked patients to mark the point on the line which they represented their current state of their symptoms. Nurses determined the VAS score by measuring in millimeters from the left-hand end to the point that patients had marked. Blood samples were collected by the medical doctors and measured by a contracted research company. Blood concentrations of dioxins, PCBs and PCQ were measured by Fukuoka Institute of Health and Environmental Sciences, Fukuoka, Japan. The detailed method to measure the organochlorine compounds is described by Todaka et al. [5].

### 2.4. Sample Size

Due to the limited number of Yusho patients, two formulae were administered to each patient for half-a-year each. Sample size could not be statistically defined, because no background information was available on the VAS for the symptoms in Yusho patients. The maximum number of eligible patients was estimated to be 100, considering the location of residence and the health condition of patients. The prevalence of general fatigue, skin symptoms, neurological symptoms and respiratory symptoms was estimated to be 60, 30, 50 and 60%, respectively, by annual medical health check-ups from 2001 to 2003 for Yusho patients [6].

### 2.5. Selection and Allocation of Formulae

When a candidate was recruited, the minimum information sufficient for the allocation was sent to one of the authors (S. Tokunaga). If the patient complained of more than two symptoms, two symptoms were selected according to severity of the symptoms, request of the patients and evenness of the number of formulae to be allocated. The order of the symptoms for which the Kampo formulae were prescribed was allocated by the minimization method stratified by age (<70/≥70 years), residency (Fukuoka, Nagasaki or Hiroshima prefecture) and sex (male/female).

### 2.6. Statistical Methods

Analysis of the primary endpoint, the effectiveness of the Kampo formulae measured by the changes in VAS from the baseline value, was evaluated by the Student's *t*-test. The effectiveness of the formula on the target symptom was compared with that of other formulae on the same symptom. In the analyses of secondary endpoints, an analysis of variance (ANOVA) with Bonferroni correction was performed on the effectiveness of Kampo formulae on symptoms other than the target symptom. The influence of Kampo formulae on patients' QOL was evaluated by ANOVA with Bonferroni correction. The concentrations of dioxins, PCBs and PCQ were log-transformed because of highly discrete distribution before applying ANOVA with Bonferroni correction. The concentrations of 1,2,3,7,8-pentachlorodibenzo-*p*-dioxin (penta-CDD), 1,2,3,4,7,8-hexa-CDD, 1,2,3,6,7,8-hexa-CDD, 1,2,3,7,8,9-hexa-CDD, 1,2,3,4,6,7,8-hepta-CDD, octa-CDD, 2,3,7,8-tetra-CDF, 2,3,4,7,8-penta-CDF, 1,2,3,4,7,8-hexa-CDF, 1,2,3,6,7,8-hexa-CDF, 3,3′,4,4′,5-penta-CB (#126), and 3,3′,4,4′,5,5′-hexa-CB (#169) were statistically analyzed. The concentrations of 2,3,7,8-tetra-CDD, 1,2,3,7,8-penta-CDF, 2,3,4,6,7,8-hexa-CDF, 1,2,3,7,8,9-hexa-CDF, 1,2,3,4,6,7,8-hepta-CDF, 1,2,3,4,7,8,9-hepta-CDF, octa-CDF, 3,4,4′,5-tetra-CB (#81) and 3,3′,4,4′-tetra-CB (#77), however, were not analyzed because the concentrations of those congeners in more than half of the patients were under the detection limit. A two-tailed *P* < .05 was considered statistically significant. All statistical tests were performed with Stata 10.1 (Stata Corp., College Station, TX, USA).

## 3. Results

### 3.1. Patient Enrollment

**Figure 1:**
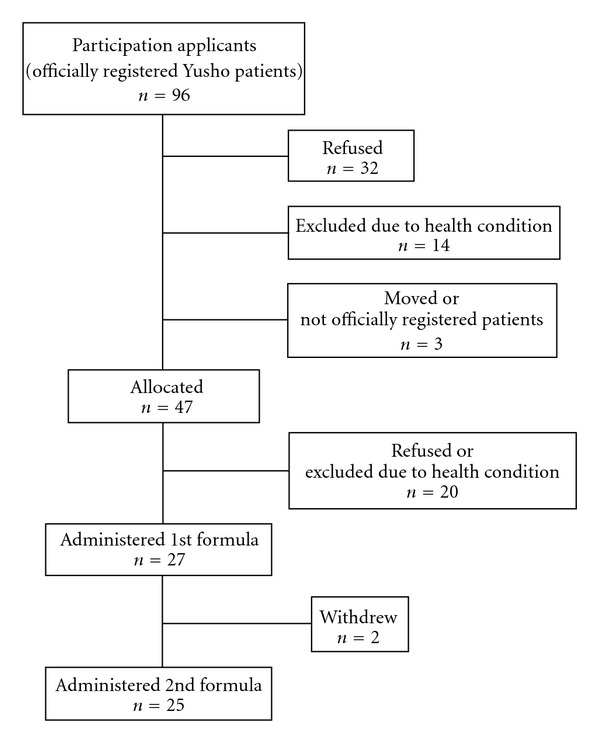
Flow of patient through the study.

The flowchart of patients through the study is shown in Figure 1. Patients were first recruited at explanatory meetings conducted in three regions of Fukuoka and Nagasaki prefectures. A questionnaire was distributed at the meetings to inquire about participation in the trial. Eighty-five patients answered the questionnaire. After the meeting, an additional 11 patients inquired about participation in the trial after getting information from various sources, such as newspapers. Of the 96 patients who inquired about the trial, 32 refused to participate and 14 were excluded due to their health condition. Another was a potential Yusho patient who was not officially registered, and two others moved and were unable to be contacted. Of the remaining 47 patients to whom two formulae were allocated, 20 could not enter the trial because of illness found after a detailed health check, the difficulty of receiving regular health check ups and refusal to participate. The remaining 27 patients participated in the trial. 

Registration of patients followed by the first treatment occurred from October 26, 2005 to October 12, 2006. During the first treatment, the number of patients prescribed with Hochu-ekki-to, Keigai-rengyo-to, Gosha-jinki-gan and Bakumondo-to, was 5, 8, 8 and 6, respectively. One patient prescribed with Hochu-ekki-to and one with Gosha-jinki-gan withdrew from the trial after the first administration.

The second treatment started on April 12, 2006 and ended on March 29, 2007. During the second treatment, Hochu-ekki-to, Kaigai-rengyo-to, Gosha-jinki-gan and Bakumondo-to were administered to 9, 5, 5 and 6 patients, respectively. The second treatment ended between September 28, 2006 and October 18, 2007. In total, Hochu-ekki-to, Kaigai-rengyo-to, Gosha-jinki-gan and Bakumondo-to were administered to 14, 13, 13 and 12 patients, respectively. Details of the combination of formulae administered to each patient were shown in Table 1. 

### 3.2. Background of the Participants

Of the 27 patients aged 51–86 years (mean = 69.4, standard deviation (SD) = 7.9) who participated in this trial, 16 were men (59.3%) and 11 were women (40.7%). They were from Fukuoka (10 patients), Nagasaki (8 patients) and Hiroshima (9 patients) prefectures. The geometric mean blood concentrations (pg/g lipid) (5 to 95 percentiles) of dioxins and PCBs were 11.8 (9.3, 16.3) for 1,2,3,7,8-penta-CDD, 2.8 (2.3, 3.7) for 1,2,3,4,7,8-hexa-CDD, 46.0 (31.1, 76.8) for 1,2,3,6,7,8-hexa-CDD, 3.6 (2.9, 5.5) for 1,2,3,7,8,9-hexa-CDD, 43.3 (29.6, 54.5) for 1,2,3,4,6,7,8-hepta-CDD, 702.2 (456.8, 987.9) for octa-CDD, 1.7 (1.3, 3.1) for 2,3,7,8-tetra-CDF, 132.9 (78.5, 385.1) for 2,3,4,7,8-penta-CDF, 29.5 (15.2, 54.2) for 1,2,3,4,7,8-hexa-CDF, 15.3 (8.1, 20.8) for 1,2,3,6,7,8-hexa-CDF, 101.4 (68.2, 172.8) for 3,3′,4,4′,5-penta-CB (#126) and 198.9 (160.7, 261.5) for 3,3′,4,4′,5,5′-hexa-CB (#169). The geometric mean (5 to 95 percentile) blood concentration for PCQ was 0.6 (0.0, 4.4) ng/g.

### 3.3. Effectiveness on Clinical Symptoms

**Figure 2:**
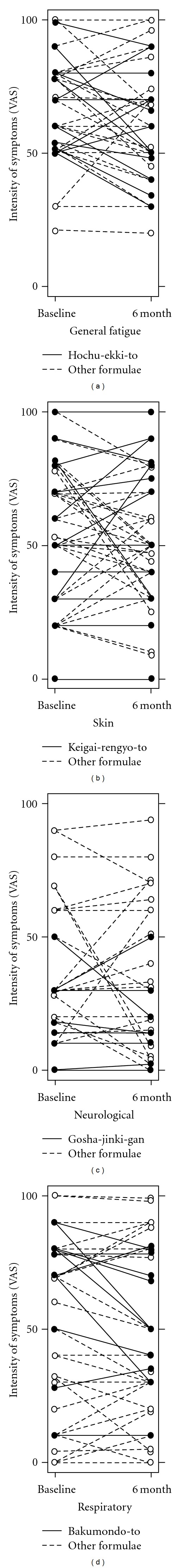
Changes in the intensity of symptoms by Kampo formulae in each patient. The intensity of symptoms was measured by VAS at the baseline and after treatment of 6 months. The values of the same patient were connected by the solid line if the symptom was targeted by the formula, or by the dashed line otherwise.

**Figure 3:**
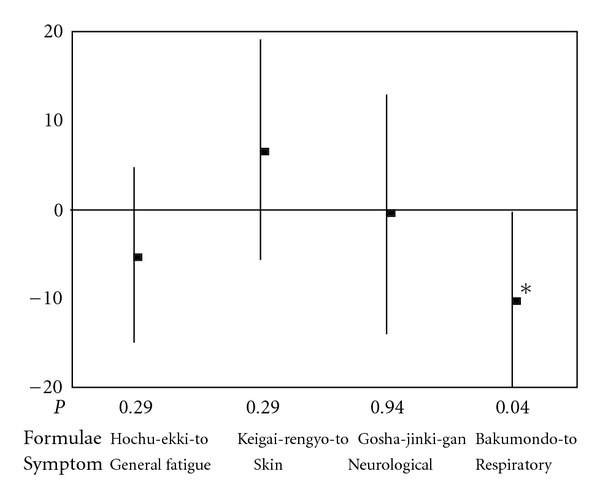
The effectiveness of Kampo formulae on the target symptom; the primary endpoint. The target symptoms were general fatigue for Hochu-ekki-to, skin symptoms for Keigai-rengyo-to, neurological symptoms for Gosha-jinki-gan and respiratory symptoms for Bakumondo-to. The effectiveness of the Kampo formulae was evaluated by changes in intensity of the symptoms from baseline to the end of administration, which was measured by VAS. Differences in effectiveness between the formulae administered for the target symptom and other formulae for the same symptom are shown. Data are means (dots) and 95% confidence intervals (bars). Bakumondo-to significantly improved respiratory symptoms compared with other formulae. *P < .05 by Student's *t*-test.

**Figure 4:**
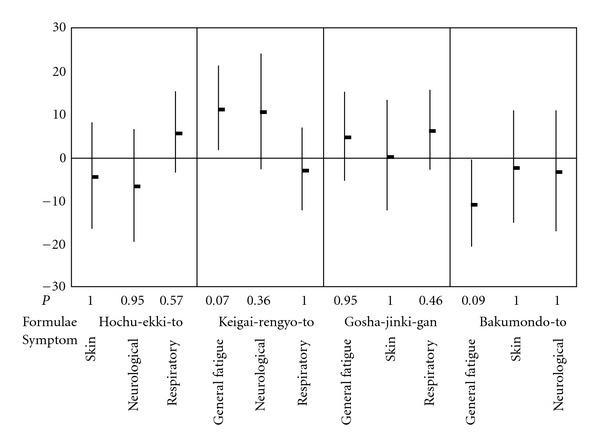
The effectiveness of Kampo formulae on symptoms other than the target; the secondary endpoints. The effectiveness of the Kampo formulae was evaluated by changes in intensity of the symptoms from baseline to the end of administration, which measured by VAS. Differences in effectiveness between the formulae for symptoms other than the target symptom are shown. Data are means (dots) and 95% confidence intervals (bars). The effectiveness of the formulae was not statistically significant, except for Keigai-rengyo-to, which worsened general fatigue by a marginal significance (*P* =  .07 by ANOVA with Bonferroni correction).

Changes in the intensity of symptoms measured by VAS from baseline to the end of administration of formulae in each patient were presented in Figure 2.  Figure 3 shows the differences in effectiveness measured by VAS between the Kampo formulae prescribed to the target symptom and the formulae primarily prescribed to other symptoms. The differences in effectiveness on general fatigue, skin and neurological symptoms between Kampo formula primarily prescribed to those symptoms and other formulae were not statistically significant. Bakumondo-to, which was administered to 11 patients, alleviated respiratory symptoms, as demonstrated by a mean change of −12.2 (SD = 17.6) on the intensity of symptoms. On the other hand, respiratory symptoms of patients administered other Kampo formulae had a decreased VAS mean change of −1.9 (SD = 13.2). This difference in effectiveness was 10.3 (95% confidence interval: 0.5–20.0) and was statistically significant (*P* =  .04). The changes in VAS after administration of the Kampo formulae for symptoms other than the target symptom are compared with each other as secondary endpoints in Figure 4. The effectiveness of the formulae was not statistically significant, except for Keigai-rengyo-to, which worsened general fatigue by a marginal significance (*P* =  .07).

### 3.4. Influence on QOL

**Figure 5:**
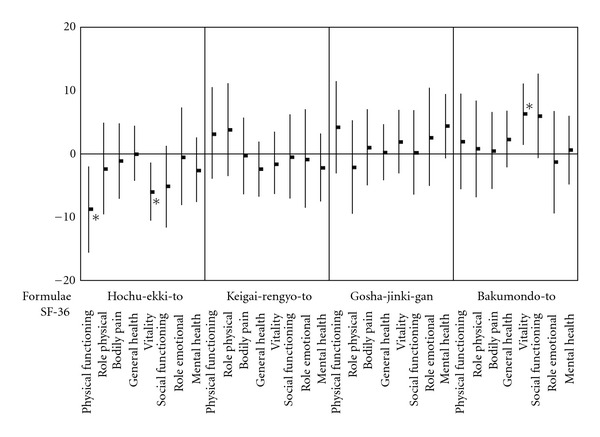
Changes in SF-36 from baseline values after administration of Kampo formulae. The influence of the Kampo formulae on patients' QOL was evaluated by changes in SF-36 from baseline to the end of administration. Differences in influence on the scores of SF-36 between the formulae are shown. Data are means (dots) and 95% confidence intervals (bars). Bakumondo-to significantly improved patients' QOL in the context of vitality, whereas Hochu-ekki-to significantly impaired patients' QOL in the context of physical functioning and vitality, compared with other formulae. *P < .05 by ANOVA with Bonferroni correction.

The baseline scores on the SF-36 (mean (SD)) were 36.9 (14.3) for physical functioning, 34.9 (10.8) for role physical, 38.0 (10.2) for bodily pain, 38.3 (9.8) for general health, 41.8 (9.5) for vitality, 39.3 (10.4) for social functioning, 35.5 (12.5) for role emotional, and 42.4 (9.3) for mental health. The comparison of changes in patients' QOL from baseline after administration of Kampo formulae is shown in Figure 5. Physical functioning and vitality scores on the SF-36 decreased after administration of Hochu-ekki-to compared to changes in SF-36 scores after administration of other formulae (*P* =  .05  and  .03, resp.). After treatment with Bakumondo-to, the vitality score on the SF-36 increased compared to the scores after administration of other formulae (*P* =  .04). These results indicated that Bakumondo-to improved patients' QOL in the context of vitality, whereas Hochu-ekki-to impaired patients' QOL in the context of physical functioning and vitality, compared with other formulae. 

### 3.5. Influence on Blood Dioxins, PCBs, PCQ and Laboratory Values of Blood

The comparison of changes in blood concentrations of dioxins, PCBs and PCQ did not show any statistically significant differences between the Kampo formulae, except for 1,2,3,6,7,8-hexa-CDF, which was not a causative toxic compound for Yusho (data not shown). The concentration of 1,2,3,6,7,8-hexa-CDF decreased by 22% compared to other formulae (*P* =  .01) after administration of Gosha-jinki-gan. The mean (min–max) number of laboratory tests for each patient was 6.7 [2–9, 11–13]. Less than 20% of observed values were outside the range of ± 15% of the standard range of each hematological component, except for triglycerides and blood sugar (data not shown).

## 4. Discussion

In the present study, Bakumondo-to significantly improved respiratory symptoms (cough and expectoration of sputum) of Yusho compared with other formulae. Bakumondo-to also improved patients' QOL in the context of vitality. On the other hand, Keigai-rengyo-to did not improve skin symptoms of Yusho, but marginally worsened general fatigue compared with other formulae. Hochu-ekki-to did not reduce general fatigue in Yusho patients, but impaired patients' QOL in the context of physical functioning and vitality compared with other formulae, suggesting that it may not be appropriate to prescribe Keigai-rengyo-to and Hochu-ekki-to for Yusho patients. Gosha-jinki-gan did not demonstrate any influence on the symptoms of Yusho. In this study, patients were administered two Kampo formulae for half-a-year each. Although we did not observe any unusual effects by any combination of formulae, and Bakumondo-to constantly improved respiratory symptoms irrespective of combinations, contaminations of other formulae to the effectiveness on the target symptom would not be excluded completely, because statistical analyses were not available due to very limited number of patients in each combination. Additionally, none of the Kampo formulae produced serious adverse effects, and blood concentrations of dioxins and related compounds were little affected by the Kampo formulae. Although 40 years have passed since the outbreak of Yusho, and most patients still suffer from various symptoms [6], no effective treatment for Yusho has been developed. Because blood concentrations of dioxins in Yusho patients significantly correlated with the intensity of Yusho symptoms [6–8], medicines that accelerate excretion of dioxins or convert dioxins to harmless metabolites could provide a permanent cure for Yusho. Yoshimura et al. [17] extensively investigated methods to accelerate the excretion of such organochlorine compounds in an animal model. They demonstrated that in rats the oral administration of different antidotes (squalene, paraffin, cholestyramine and charcoal) increased the excretion of 2,3,4,7,8-penta-CDF 2–5 times more than amounts excreted in the control group [17, 18]. In addition to these antidotes, it was found that dietary fiber accelerated the excretion of congeners of polychlorinated biphenyls [19]. Based on these studies, cholestyramine and rice bran fiber were administered for 2 weeks to a small number of patients with Yusho and Yucheng, another poisoning caused by ingestion of oil contaminated with dioxins. The amounts of 2,3,4,7,8-penta-CDF in their feces actually increased, although beneficial clinical effects were not apparent, possibly due to the short trial period [20].

Another alternative treatment for Yusho would be antagonistic compounds against the aryl hydrocarbon receptor (AhR), which is well known as a receptor for dioxins and other polycyclic aromatic hydrocarbons [21]. The AhR is a ligand-activated transcription factor that is ubiquitously distributed in the body, and after ligation of dioxins to the AhR, the receptor translocates from the cytosol to the nucleus, heterodimerizes with the AhR nuclear translocator (ARNT), and binds to an enhancer sequence, called a dioxin response element (DRE), of several drug metabolizing enzymes, such as cytochrome P450 1A1 (CYP1A1) [21]. Previous investigations using AhR-null mutant mice confirmed a critical role of the AhR in the toxic effects of dioxins [22, 23]. The present functional role of the AhR was derived mainly from recent studies using environmental contaminants such as dioxins. Therefore, the AhR might primarily be a regulatory receptor for exogenous natural products such as food constituents. Various polyphenol compounds that are present in vegetables, fruits and herbs have been shown to have antagonistic potencies against the AhR. Flavones (apigenin, luteolin, baicalein), flavonols (quercetin, kaempferol, myricetin), flavanones (naringenin, hesperetin), anthraquinones (emodin, alizarin), curcumin, resveratrol and coumestrol have strong inhibitory potencies against AhR activation induced by 2,3,7,8-tetra-CDD [24–27]. Flavonoids belonging to the flavone, flavonol and flavonone subclasses directly bind to the AhR complex in the cytosol, and suppress AhR transformation by competitive inhibition of binding between the AhR and its agonist [28]. In this study, the effectiveness of the Kampo formula on the target symptom was compared with that of other formulae on the same symptom. We hypothesized that the formulae might have not only expected effects for target symptoms but also antagonistic activities on dioxins by unknown ingredients. We supposed that we would find formulae having anti-dioxin activities by comparing the effectiveness of the formula to the target symptom with other formulae on the same symptom.

**Figure 6:**
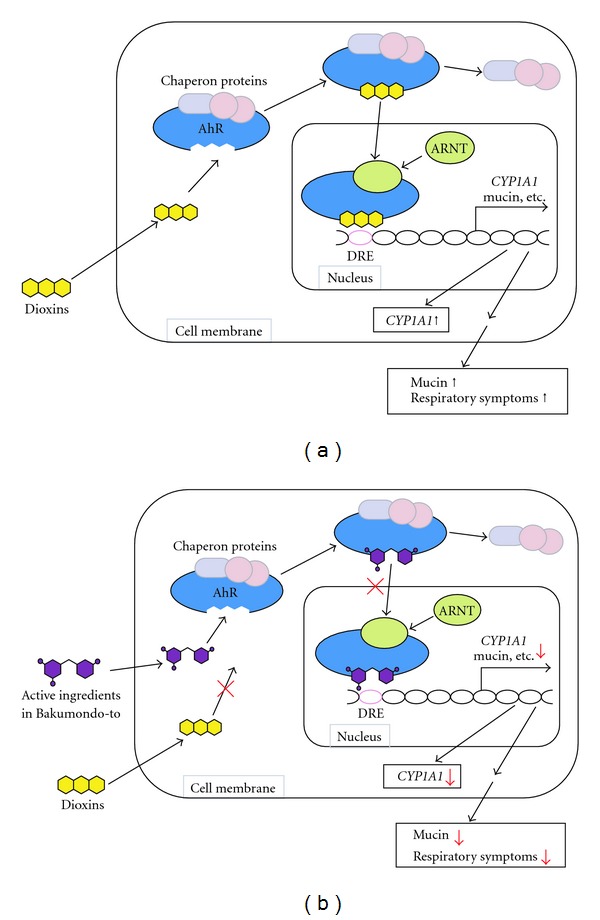
Hypothetical diagrams of the effect of Bakumondo-to. (a) After ligation of dioxins to the AhR, the receptor translocates from the cytosol to the nucleus, binds to DRE, and activates transcription of *CYP1A1*, mucin and other genes in bronchiolar Clara cells. Excess mucin production from Clara cells by dioxins is supposed to be responsible at least in part for respiratory symptoms of Yusho patients. (b) Active ingredients in Bakumondo-to would block activation of the AhR by competitive interference with the interaction between dioxins and the AhR, and inhibit transcription of mucin in Clara cells, leading to improvement of respiratory symptoms of Yusho patients.

The intensity of respiratory symptoms, such as cough and expectoration of sputum, were shown to be significantly correlated with blood concentrations of dioxins in Yusho patients [7]. In addition, bronchiolar Clara cells and ciliated cells constitutively express the AhR, and dioxins such as 2,3,7,8-tetra-CDD and poly-CDFs impaired Clara cells in the lung of rats [29, 30]. Recently, 2,3,7,8-tetra-CDD was shown to induce mucin production from a Clara cell-derived cell line [31]. Therefore, activation of the AhR by dioxins could cause the respiratory symptoms in Yusho patients. Although further studies would be needed to clarify how Bakumondo-to improves respiratory symptoms of Yusho, active ingredients in Bakumondo-to might interfere with the interaction between dioxins and the AhR in bronchioles, and improve respiratory symptoms in Yusho patients. Although we have not identified what constituents in Bakumondo-to are effective yet, a hypothetical diagram is shown in Figure 6. We are now conducting basic studies to investigate the mechanism by which Bakumondo-to and its constituents affect the function of the AhR.

In conclusion, we have demonstrated for the first time that a Kampo formula, Bakumondo-to, significantly improved respiratory symptoms (cough and expectoration of sputum) of Yusho compared with other formulae. This formula may be effective for the treatment of respiratory symptoms of patients intoxicated with other dioxins, such as 2,3,7,8-tetra-CDD.

## Figures and Tables

**Table 1:** Combination of Kampo formulae administered to each patient.

	Second formula					
	Hochu-ekki-to	Keigai-rengyo-to	Gosha-jinki-gan	Bakumondo-to	(Withdrew)	Total
First formula						
Hochu-ekki-to	—	1	3	0	1	5
Keigai-rengyo-to	4	—	1	3	0	8
Gosha-jinki-gan	4	0	—	3	1	8
Bakumondo-to	1	4	1	—	0	6

Total	9	5	5	6	2	27
